# Editorial: Molecular pathogenesis and novel treatments for inherited cardiomyopathies

**DOI:** 10.3389/fcvm.2023.1282852

**Published:** 2023-09-06

**Authors:** Chun Chou, Hilary J. Vemon, John L. Jefferies, Michael T. Chin

**Affiliations:** ^1^ Department of Medicine, Tufts University School of Medicine, Boston, MA, United States; ^2^ Department of Genetic Medicine, Johns Hopkins University School of Medicine, Baltimore, MD, United States; ^3^ Division of Cardiology, Department of Medicine, University of Tennessee Health Sciences Center, Memphis, TN, United States; ^4^ Molecular Cardiology Research Institute, Tufts Medical Center, Boston, MA, United States

**Keywords:** inherited cardiomyopathy, hypertrophic cardiomyopathy, dilated cardiomyopathy, iron overload cardiomyopathy, rasopathy, arrhythmogenic cardiomyopathy

**Editorial on the Research Topic** Molecular pathogenesis and novel treatments for inherited cardiomyopathies

Inherited cardiomyopathies have diverse genetic etiologies, but together are major contributors to cardiac disease that affect patients of all ages, typically with early onset in childhood or adolescence. While inherited cardiomyopathies are currently classified according to functional and morphologic features, finer resolution of these categories has been made possible with the aid of modern genetics. However, despite the identification of many disease-causing variants, effective therapies for the majority of inherited cardiomyopathies remains scarce. In this issue, a collection of original research and review articles unveil and summarize the pathogenic mechanisms and gene expression signatures of various inherited cardiomyopathies as well as novel and extant therapeutic strategies in treating selected subsets of inherited cardiomyopathies.

While genetic evaluation of family with a cardiomyopathy is increasingly common, in clinical practice, molecular insights do not supersede the clinical phenotyping and classification of inherited cardiomyopathies since different mutations in the same gene can result in distinct disorders. This is best exemplified in a review by Hilal et al. which discusses in detail how mutations in RAS genes underlie phenotypically diverse cardiomyopathies in addition to distinct systemic syndromology. Notably, one must cautiously distinguish cardiomyocyte-intrinsic from extrinsic effects of RAS hyperactivation. While several *in vitro* studies demonstrated that ectopic RAS activation results in cardiomyocyte hypertrophy, cardiomyocyte-specific expression of gain-of-function RAS variants did not result in HCM despite cardiomyocyte hypertrophy seen in neonatal mice carrying the same germline mutation in the RAS gene (1). Whether other RAS variants promote cardiomyopathy in a cardiomyocyte-extrinsic mechanism has not been exhaustively tested.

As the affordability of multiple “omics” technologies have increased in recent years, whole-exome sequencing has become the new standard to identify novel genetic variants. Recently, a variant of crystallin alpha B, R123W, was identified in a pair of homozygotic twins who develop concordant HCM (2). Chou et al. further demonstrated that mice carrying the *Cryab*^R123W^ indeed recapitulates several key features of human HCM. Mechanistically, this CRYAB variant promotes pathologic calcium signaling through an unexpected protein-protein interaction with calcineurin, uncovering yet another sarcomere-independent mechanism of HCM pathogenesis. Also included in this issue was a case report by Liu et al. who identified a novel variant of DSG2 in a pediatric patient with arrhythmogenic cardiomyopathy misdiagnosed as myocarditis. Although both CRYAB^R123W^ and DSG2 c.1592T > G are rare variants, they nevertheless provided novel insights into previously unappreciated pathogenic mechanisms of inherited cardiomyopathies.

In addition to mutations in protein-coding genes, non-coding genetic variants have increasingly been recognized as potential modifiers or primary contributors towards pathogenic processes. Htet et al. reviewed known associations between non-coding genetic mutations and various inherited cardiomyopathies. In addition to splice site variants which affect function of encoded protein, mutations in other regulatory elements, such as untranslated regions and microRNAs, are also reviewed. Cao and Yuan further uncovered that three distinct lncRNAs were differentially expressed between control and HCM patients regardless of sarcomere mutation. Intriguingly, expression of mRNA co-expressed with these lncRNA is not consistently different between HCM patients and controls. Thus, the mechanism by which the three lncRNAs are differentially regulated during HCM pathogenesis is unclear. As with other omics studies, whether differential expression of lncRNA is biologically relevant remains to be tested.

In addition to identifying genetic mutations associated with various inherited cardiomyopathies, mechanistic studies that elucidate the molecular consequences of these genetic mutations are also underway. Considering that adult hearts almost solely utilize fatty acid oxidation to generate ATP, Gaar-Humphreys et al. reviewed how biological processes associated with lipid metabolism are altered in various inherited cardiomyopathies. Intriguingly, disrupted metabolism is present in inherited cardiomyopathies regardless of the pathogenic DNA variant or phenotypes, though etiology- and phenotype-specific alterations in lipid metabolism also exist. Notably, expression of PPAR family transcription factors, key regulators of fatty acid uptake and oxidation, is generally suppressed in both DCM and HCM. Although how PPAR affects DCM and HCM pathogenesis remains unclear, several well-established PPAR agonists are readily available for pre-clinical studies.

Lastly, a series of seminal studies and reviews summarize recent advances in diagnostic tools and medical therapies for various inherited cardiomyopathies. While increasing evidence suggest that additional etiologies, aside from the canonical monogenic mechanism, for HCM are prevalent, sarcomere gene mutations leading to contractile dysfunction still represents a prevailing model of HCM pathogenesis. Extensive translational research over the past decades has thus focused on developing therapies to reverse sarcomere defects. A mini review by Sewanan and Shimada summarizes the results of clinical trials assessing efficacy of myosin-targeted agents, including mavacamten in HCM patients with LVOT obstruction. Whether mavacamten also delays cardiac remodeling in patient without LVOT obstruction is currently being tested. Furthermore, considering that cardiomyocyte hypertrophy is a defining feature of HCM regardless of sarcomere gene mutations, it would be intriguing to evaluate whether myosin-modulators also exert similar beneficial effect in patients without sarcomere mutations, using subgroup analysis of existing trials.

The structural changes associated with cardiac remodeling predispose HCM patients to ventricular arrythmias. However, there has not been a practical biomarker to predict who is most at risk of developing arrythmia. Zhang et al. analyzed EKG patterns in a cohort of Chinese population with HCM who has also received ICD implantation, and identified QTc duration > 464 ms and long or deep S wave in V4 lead as predictive markers for appropriate ICD shocks. Future retrospective studies to assess how well these EKG abnormalities predict first ventricular arrythmias in patients without ICD as secondary prevention would be valuable in guiding timing of ICD implantation.

Considering that diagnosis of DCM is often delayed until signs and symptoms of heart failure manifest, Zhang et al. performed multi-model machine learning on existing microarray data from DCM patients and healthy controls to develop a predictive model that may facilitate early DCM diagnosis. Notably, three DCM signature genes were identified and reassuringly, the plasma levels of proteins encoded by all three genes were indeed altered in an independent patient cohort. It is, however, surprising that three genes are sufficient to identify DCM in a testing dataset. A few caveats remain: the severity of disease in patients from the testing dataset is unclear and whether the “healthy controls” contain patients with undiagnosed DCM also remains unclear. Additionally, whether the expression pattern of these three genes is specific to DCM needs to be tested.

Another form of inherited cardiomyopathy, iron overload cardiomyopathy (IOC), represents a major co-morbidity of genetic hemochromatosis as well as secondary iron overload. A recent clinical trial demonstrated that amlodipine and chelation combination therapy significantly lowers intracardiomyocyte iron deposition. Mechanistically, amlodipine appears to block the L-type calcium channel, which otherwise provides a major route for iron entry into the cardiomyocyte. Zhabyeyev et al. demonstrated that a genetic mouse model of primary hemochromatosis when fed with high iron diet recapitulates histological, electrical and echocardiographic defects seen in patients with primary hemochromatosis. Furthermore, such phenotypes were markedly reversed with amlodipine treatment. Although the study does not provide further mechanistic insights, it nevertheless highlights a translatable mouse model that may serve as platform for development of future IOC therapies.

Over the past decades, the identification of genes in which pathogenic variants are associated with inherited cardiomyopathies has raised expectations for new therapies. Advances in genomic editing technology hold promise for directly targeting pathogenic variants, and have the potential for shifting the paradigm for treatment in genetic medicine. Other approaches harnessing natural genetic modifying mechanisms that suppress the penetrance of pathogenic variants may also be useful. While the incomplete penetrance of inherited cardiomyopathies often complicates the genetic evaluations of families with cardiomyopathies, the observation that many mutation carriers remain disease free in fact provides paradoxical hope that disease-modifying therapeutics may be achievable. Future omics studies comparing transcriptome and proteome between clinically active and silent mutation carriers may thus uncover novel therapeutic approaches to delaying disease onset. In parallel, development of additional genetic animal models that approximate human cardiomyopathies will enable rigorous mechanistic studies that potentiate development of therapeutic agents.
